# A Child with a Painful Foot: How to Get a Definitive Diagnosis

**DOI:** 10.1155/2022/5105078

**Published:** 2022-03-08

**Authors:** R. J. L. L. van de Kimmenade, D. T. Meijer, D. Hoornenborg, H. M. van der Vis

**Affiliations:** ^1^Department of Orthopaedics and Traumatology, Tergooi Hospital, Riebeeckweg 212, 1213 XZ Hilversum, Netherlands; ^2^Xpert Clinics Orthopedie Amsterdam, Laarderhoogtweg 12, 1101 EA Amsterdam-Zuidoost, Netherlands

## Abstract

This case report describes an 8-year-old healthy boy with a retained wooden foreign body in his 4^th^ metatarsal bone of his right foot. He was presented several months after the initial trauma, stepping into a toothpick, had occurred. He was operated in our hospital, and the wooden toothpick was removed. The operation and recovery were both uncomplicated. History and physical examination are essential in these types of cases with a history of penetrating trauma but can be inconclusive due to the delay in presentation. Aggressive treatment on the other hand is necessary to prevent bigger problems such as the development of inflammatory problems and persisting complaints. A thorough, systematic, and complete work-up from the history to treatment is therefore necessary and will be described in this case report.

## 1. Introduction

Puncture wounds of the foot, with or without a retained foreign body, are commonly seen by general practitioners and on emergency departments in hospitals. A thorough and systematic approach of a patient with this kind of trauma is essential and prevents more complex problems. In the case of foreign bodies of organic origin, such as wood, cellulitis, osteomyelitis, abscess formation, and pseudotumor formation can occur [1].

In this case report, we present a patient with a retained wooden foreign body in the foot. We will describe the difficulties and try to give a summary and structure how to do a proper work-up.

## 2. Case Presentation

An 8-year-old healthy boy was seen on our outpatient clinic with complaints of his right foot. For several months, he had been complaining about pain in his forefoot. Initially, the pain was intermittent and only with prolonged exertion. During the last two months, the pain was continuous and increased with load bearing. The constant pain was aching in nature and became sharp under load. During the night, there were no real complaints. Sometimes, the foot was also swollen, especially after playing soccer. Limping was present in the beginning, but after several days, this disappeared. The patient and his parents could not remember a trauma of the foot, especially no penetrating trauma. With this story, he was referred to our hospital by the general practitioner.

An orthopedic surgeon performed a thorough physical examination. Inspection revealed no suspect findings; there were no skin abnormalities on the plantar and dorsal side of the foot suggesting a penetrating trauma. There was a full and painless range of motion in the ankle, subtalar, Chopart, and Lisfranc joint. The 4^th^ metatarsal bone was painful when palpated both plantar and on the dorsal side. The lower extremity was neurovascular intact.

**Figure 1:**
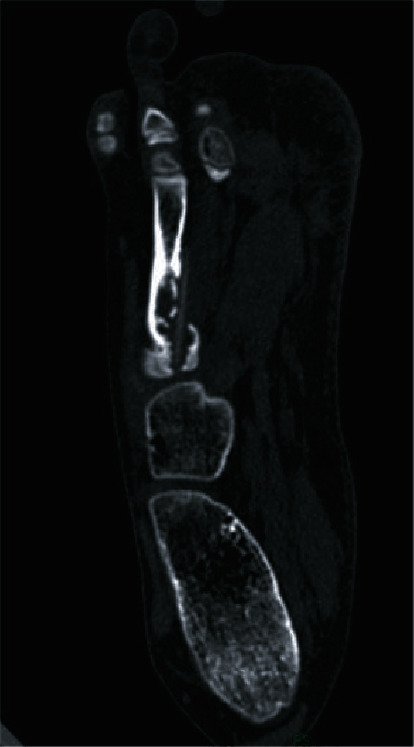
Transversal computed tomography key image with a linear hyperintense configuration, suggesting the presence of a foreign body.

Radiographs of the foot showed no significant abnormalities. There we no signs of an earlier fracture; the cortical bone was intact, and no callus formation was seen. In the medullary cavity, there were no abnormalities. The radiologist or we did not recognize a foreign body. An additional computed tomography (CT) scan was made to get more information. On the transversal images, there was a linear hyperintense configuration, seen from the proximal intramedullary end of the 4^th^ metatarsal continuous to distally and on the medial site into the soft tissue. On the medial side, the cortex was interrupted as well. These findings suggested the presence of foreign body (Figure 1). Eventually, the parents remembered a situation where our patient was walking bare feet during the summer when the family was having a barbecue; this was 8 months before his presentation on our outpatient clinical ward. During this barbecue, he suddenly cried and went to his parents with pain of his foot.

**Figure 2:**
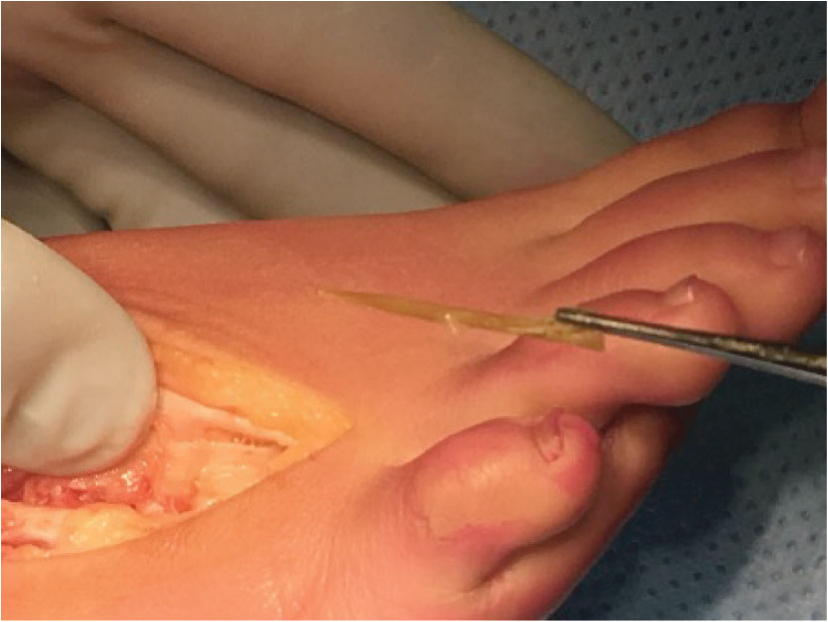
Peroperative taken clinical picture, with the completely removed wooden foreign body.

The patient was operated, and preoperative, an ultrasound was made to mark the location of the foreign body. A dorsal incision was made at the marked place. Great care was taken to prepare the metatarsal bone, taking into account the neurovascular bundles and the extensor tendon. On the medial side, a small bone hatch was made to open the intramedullary cavity. The wooden foreign body was detected and removed completely. It had the shape of a toothpick (Figure 2). The bone hatch was repositioned, and the periost was closed with Vicryl 2.0; the skin was closed with Vicryl Rapide.

Postoperative treatment consisted of a cast for one week, followed by weight bearing on a DARCO shoe^®^. The wound healed without complication. Six weeks postoperatively, the patient was walking and playing soccer without pain. We discharged him from further follow-up.

## 3. Discussion

In general, a proper history and physical examination of every patient give the best information, whatever the problem is. In our case, there was a delay in the presentation, which can make a complete history difficult. When it concerns a child, limping is a red flag. A systematic approach is necessary to prevent mis- or underdiagnosis. Transient synovitis of the hip is the most common diagnosis for a (sub) acutely limping child [2]. Other acute causes of an acute limping child include a contusion, foreign body in the foot, fracture, osteomyelitis, septic arthritis, and reactive arthritis (Table 1). Evaluation of the limping child should begin with a history focused on identifying pain, trauma, and associated systemic symptoms [2].

Important during the history is to obtain particular information. When did the injury occur? Did the patient see what the penetrating object was? Was the patient wearing footwear at the time of injury? Essential is to update the patient's tetanus status [2, 3].

Obtaining a complete history is often hard, due to the fact that patients are often presented for evaluation several months or even years after the initial trauma occurred [3]. Consequently, due to this delay, clinical evaluation may fail to elicit of antecedent skin puncture. Some describe that there is an inherent danger of relying solely on the clinical examination as a missed foreign body on initial examination may be close to 38% [4].

During physical examination of the foot after a puncture wound, the examiner must first carry out a neurologic and vascular examination. The toes are assessed for mobility to determine whether a tendon laceration has occurred. Careful attention is needed to differentiate between the long and the intrinsic flexors of the foot [5].

Then, the puncture wound itself has to be evaluated, especially if it concerns a recent trauma. Examiners need to check whether there is a retained foreign body but also look for symptoms matching with local infection. Crepitus on palpation of the soft tissue may indicate deep infection with abscess or subcutaneous gas [2, 5].

The detection of a wooden foreign body is essential because it is an excellent medium for microorganisms [1, 3, 5–7]. If it is not recognized in an early stage, symptoms like cellulitis, abscess, or fistula formation could arise. When the wooden foreign body penetrates joints or bony structures, it causes synovitis or osteomyelitis, respectively [3]. Puncture wounds around the metatarsophalangeal joint or heel and the surrounding tissues often penetrate deeper because of the weight-bearing function of these areas of the foot [5].

In our case, the presentation was also delayed and only after the CT scan result did the parents remember an incident on their holiday. The specific questions about a penetrating trauma were not asked in our history, because at the time of presentation, it was not in our differential diagnosis. The physical examination was complete in our case, and the choice to carry out additional radiological examinations was justified, also in view of the duration and persistent complaints. In future cases presenting with comparable histories, retained foreign bodies should be included as differential diagnosis.

The first step is to make additional radiological examinations. Because of its availability and effectiveness in detecting radiopaque objects, metal objects as an example, plain radiographs are the initial test of choice in the investigation of foreign bodies in the foot [3, 5–7]. Organic foreign bodies, like wood, tend not to be radiopaque and usually are negative unless there is a bone reaction [3, 5]. In the differential diagnosis, one should always think of chronic osteomyelitis or bone tumors. Plain radiographs are in 85% of patients with a wooden foreign body negative, so additional imaging modalities like ultrasound and CT should always be made [7]. In our case, the plain radiographs were negative as well, due to the fact that our foreign body was made of wood.

Ultrasound is an accurate test for detection of foreign bodies and to assess potential complications, such as tendon laceration [3, 5, 8]. Especially in detection of wooden foreign bodies, ultrasound has a high sensitivity and specificity in making the diagnosis complete [3]. Given the markedly different acoustic impedance of wood and soft tissues, retained wooden foreign bodies are easily identified, with the leading edge of the echogenic wood resulting in marked acoustic shadowing [3]. Remarkable is that ultrasound often is used after the use of other modalities, such as a CT scan or MRI. When it is compared with CT and MRI scans, it is more readily available, less expensive, and superior in the detection of wooden foreign bodies, despite the size of it [5]. We only used the ultrasound for preoperative localization of the foreign body and not as a diagnostic tool. Looking at the evidence in the literature, the ultrasound is a good and cost-effective way to diagnose wooden foreign bodies. In retrospect, this will be a tool that we are going to use more often in these types of cases. So, after a plain radiograph, the ultrasound could and should be the next step in the work-up.

CT scans are also useful. In the series looked at by Peterson et al., retained wooden foreign bodies were more subtle when using the standard window level setting. When the settings are adjusted by increasing the window width, the foreign bodies are easier to find [3, 5, 6]. The attenuation of a retained wooden foreign body varies in relation to the content of air and fluid in the interstices wood. Within approximately one week, the wood absorbs blood products and exudate and increases its attenuation [3]. Also, the type of wood can be an important factor [5, 7]. We used the CT scan as additional examination after the plain radiographs; in our case, the diagnosis was partially complete. A foreign body was seen, but the material it was made of was revealed after the surgery.

The identification of wood on MRI scans can be difficult, especially when the foreign body is small and if there are no associated abscesses or fluid collections. In such cases, the foreign body may appear as a signal void with surrounding nonspecific granulation tissue [5]. Linear signal voids may be mistaken for tendons or dense collagenous structures. In the acute setting, surrounding hemorrhage and hematoma may be seen, being replaced in time with granulation tissue. Identification of this inflammatory response can assist the viewer in identifying the location of the suspected foreign body, but the foreign body itself may be difficult to visualize [7]. The surrounding foreign body reaction could also mimic a soft tissue mass or a tumor if the central foreign body is not identified [9].

Summarizing, the sequence of additional imaging when a penetrating trauma has occurred with a wooden foreign body should consist of plain radiographs, followed by an ultrasound complemented by CT scan or in some cases MRI scan if the diagnosis is not complete and clear.

## 4. Conclusion

A thorough history and physical examination is essential, especially in limping and painful children because there is a broad differential diagnosis. Ultrasound should get a more prominent role in making the diagnosis complete, and it has a good sensitivity and specificity in detecting and localizing retained wooden foreign bodies. Aggressive treatment is necessary to prevent bigger problems such as the development of inflammatory problems.

## Figures and Tables

**Table 1:** Broad differential diagnoses in the limping child. A detailed history and physical examination, appropriate laboratory tests, and imaging are essential for making a correct diagnosis.

Bone conditions	Intra-articular conditions	Soft tissue conditions	Spinal conditions	Neuromuscular conditions	Intra-abdominal conditions
- Limb length discrepancy - Developmental dysplasia of the hip - Congenitally short femur - Clubfoot - Slipped capital femoral epiphysis - Trauma (with accompanied fracture) - Osteomyelitis - Osteonecrosis (sickle cell disease) - Benign neoplasm - Malignant neoplasm	- Trauma (with accompanied fracture) - Hemarthrosis (hemophilia, after trauma) - Transient synovitis - Reactive arthritis - Acute rheumatic fever - Juvenile rheumatic fever - Septic arthritis (Lyme disease) - Congenital conditions (discoid lateral meniscus)	- Sprains and strains - Foreign body - Idiopathic tight Achilles tendon - Jumper's knee - Osgood Schlatter disease - Severe disease - Chondromalacia patellae - Cellulitis - Soft tissue abscess - Due to child abuse	- Diskitis - Vertebral osteomyelitis - Spinal cord tumors	- Cerebral palsy - Muscular dystrophy - Meningitis - Myelomeningocele	- Psoas abscess - Appendicitis - Neuroblastoma

## Data Availability

Previously reported article data were used to support this study and are available at DOI 10.3944/AOTT.2015.14.0146; 10.2214/ajr.178.3.1780557; 10.1016/0002(82)90603-1; 10.1016/j.cpm.2012.02.002; 10.7547/16-095; 10.1016/j.fas.2009.04.006; and 10.1177/1938640016656784 and PMID 26554284 and 7982157. These prior studies (and datasets) are cited at relevant places within the text as references [1–9].
